# The effect of layer thickness and immobilization chemistry on the detection of CRP in LSPR assays

**DOI:** 10.1038/s41598-022-04824-9

**Published:** 2022-01-17

**Authors:** Stephan Kastner, Pia Pritzke, Andrea Csáki, Wolfgang Fritzsche

**Affiliations:** Department Nanobiophotonics, Leibniz Institute of Photonic Technology (IPHT), Albert-Einstein-Strasse 9, 07745 Jena, Germany

**Keywords:** Biomarkers, Nanobiotechnology, Sensors and probes, Surface chemistry, Biophotonics

## Abstract

The immobilization of a capture molecule represents a crucial step for effective usage of gold nanoparticles in localized surface plasmon resonance (LSPR)-based bioanalytics. Depending on the immobilization method used, the resulting capture layer is of varying thickness. Thus, the target binding event takes place at different distances to the gold surface. Using the example of a C-reactive protein immunoassay, different immobilization methods were tested and investigated with regard to their resulting target signal strength. The dependency of the target signal on the distance to the gold surface was investigated utilizing polyelectrolyte bilayers of different thickness. It could be experimentally demonstrated how much the LSPR-shift triggered by a binding event on the gold nanoparticles decreases with increasing distance to the gold surface. Thus, the sensitivity of an LSPR assay is influenced by the choice of immobilization chemistry.

## Introduction

The C-reactive protein (CRP) is an important biomarker for inflammation and infection of the human body^1–4^. CRP is an acute phase protein of the pentraxin family formed in the liver, as a marker of general or post-operative infectious diseases primarily for bacterial infections^5^, acute myocardial infarction, and other diseases^6^. In healthy people, the concentration of CRP in serum is below 10 mg/L. Between 10–40 mg/L is typical for mild inflammation and viral infections, while active inflammation and bacterial infections result in levels of 40–200 mg/L^3^. Thus, the CRP level correlates with the stage of the diseases and is a decisive criterion for the prescription of antibiotics for the patient^7^. Therefore, diagnostic detection is very important. Additionally, CRP detection allows for a discrimination between bacterial and viral infections^8^. The most used diagnostic methods for CRP are rapid point-of-care tests (POCT) based on lateral flow-assays with a sensitivity of 10 mg/L. Though surface plasmonic resonance sensing for CRP is becoming more common there are still very few such methods close to diagnostic use^9–11^. Several plasmonic nanoparticle-based methods are established in enzyme-linked immunosorbent assay (ELISA) platforms. These assays use labeled secondary antibodies in sandwich assays^12–16^ or metal-enhanced optical signals by enzymatic deposition^15^ or metal-enhanced fluorescence^17^ for the signal enhancement.

Direct detection of CRP with plasmonic nanoparticles is possible altogether avoiding labels and secondary antibodies. A simple detection is the main advantage of colorimetric assays. The binding of CRP on the particles either stabilizes against salt-induced aggregation, or competes with bound aptamers, leading to destabilization of the nanoparticle solution^18^. Besides colorimetric detection, which yield somewhat qualitative results, plasmonic nanoparticles can also act as transducers. This is accomplished by using the change in spectroscopic properties (resonance wavelength) upon refractive index change (binding of molecules on the surface) enabling quantitative detection. Examples include the direct binding of CRP on anti-CRP antibodies (anti-CRP-AB)-modified gold nanospheres^19^, nanorods modified with single chain variable fragment (scFv)^20^, or silver nanoprisms modified with cytidine 5´-diphosphocholine (PC)^21^. In combination with straightforward optical readout units, LSPR sensors can also provide a new field of application for on-site diagnostics.

**Figure 1:**
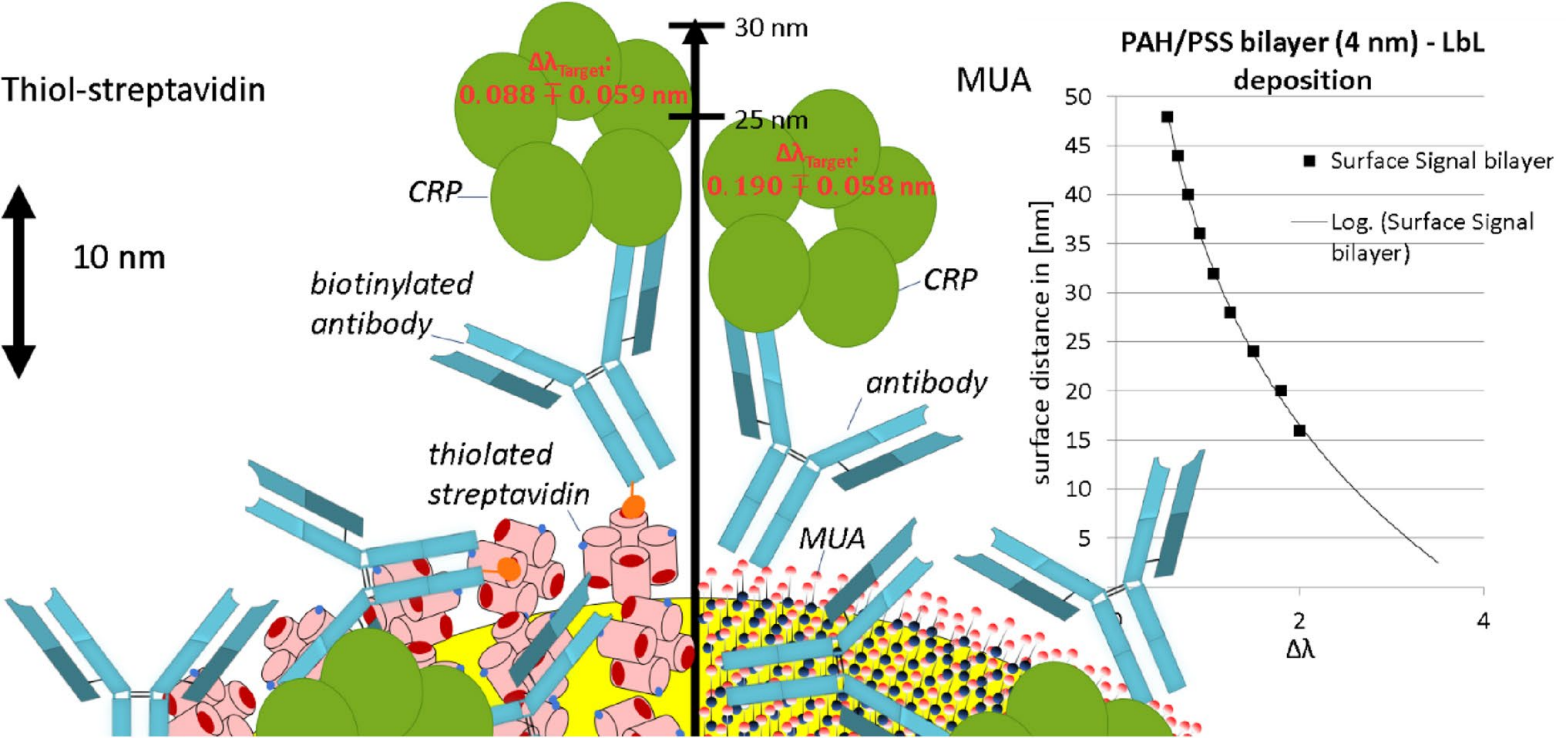
Scheme of the studied immobilization approaches for anti-CRP antibodies. Left: Biotinylated anti-CRP antibodies are attached to the gold surface by thiolated streptavidin. Right: After a surface modification by a self-assembled monolayer of MUA, EDC chemistry is utilized to attach unmodified anti-CRP antibodies. The inset shows the decrease of the sensor signal with increasing surface distance for layer-by-layer (LbL) deposition with charged polyelectrolyte (PEL) bilayers.

Because the capture-antibody-immobilization determines the critical analytical parameters such as sensitivity, reproducibility, and robustness, this step has to be adapted and optimized for a given assay on a given technological platform. In the case of LSPR, gold nanoparticles (AuNP) represent the sensors used, and have to be modified with the detection antibodies. A wide range of antibody immobilization approaches have been developed during the past few decades, starting with passively adsorbing the antibodies on the substrate, and subsequently establishing various functionalization and cross-linking strategies, overcoming certain shortcomings of earlier methods^22–24^. Two methods for this substrate preparation—immobilization of the anti-CRP antibodies on the gold nanoparticles (Fig. 1)—are investigated and compared regarding ease of use, and achieved performance e.g., as characterized by the measured signal (peak shift).

The first method studied was straightforward using thiolated streptavidin, attached to the AuNP, and able to bind biotinylated antibodies (here called SH-SA method). This method was then compared with the attachment via the well-established but more complex EDC/NHS-chemistry. The nanoparticle surface was modified with a self-assembling monolayer (SAM) of 11-Mercaptoundecanoic acid MUA (or mixed MUA layer with 1-octanethiol (1-OT) or 11-mercaptoundecanol (MUD)), attached by the thiol group to the AuNP, and exposing carboxyl groups (here called SAM method). Then, these carboxyl groups were activated by incubation with EDC and NHS to form a NHS-ester^25^, prior to incubation with the antibodies, which will bind via free amine groups to form a covalent amide bond^26,27^.

A straightforward label-free method is presented, which allows the repeated use of the sensor transducers, as chemical approaches for regeneration (removal of the target molecule from capture antibody) are demonstrated. The sensor substrate (chip) with immobilized nanoparticles is inserted in a microfluidic chamber, prior to monitoring the signal of the absorbance of the nanoparticles by a spectrometer. Different binding chemistry schemes for the attachment of the antibodies as recognition elements are compared. The defined layer-by-layer deposition is used to prepare distance layers comparable in thickness to both studied immobilization chemistries.

## Materials and methods

### Materials

Poly(allylamine hydrochloride) (PAH), polystyrene sulfonate (PSS) were purchased from Sigma-Aldrich Chemie GmbH (Taufkirchen, Germany), N-Hydroxysuccinimid (NHS), glacial acetic acid, sodium chloride (NaCl), sodium hydroxide (NaOH), glycine, 1-Ethyl-3-(3-dimethylaminopropyl)carbodiimid-hydrochlorid (EDC), ethanol, HCl, 3-triethoxysilylpropylamine (APTES), 10 × PBS Buffer and BSA were purchased from Carl Roth GmbH (Carl Roth GmbH + Co. KG, Karlsruhe, Germany). Sodium acetate was purchased from Merck KGaA (Darmstadt, Germany). Human C-reactive protein, anti-hCRP-capture-antibody biotinylated (α-hCRPcb) and non-biotinylated (α-hCRPc) and anti-hCRP-detector-antibody (α-hCRPd) was purchased from Senova GmbH (Weimar, Germany). All proteins were used as received without further purification and all used antibodies are monoclonal. Sodium acetate buffer (NaAc) was prepared by dissolving sodium acetate close to 10 mM in ultrapure water, pH was adjusted with HCl/NaOH to 4, 4.5 or 5 and filled up with ultrapure water to a final concentration of 10 mM NaAc. Glycine–HCl Buffer (Gly-HCl) was pre-pared similarly but with glycine and adjusted to pH 2.5.

### AuNP-chip preparation

Schott borosilicate wafers were cut into 25 × 16 mm glass substrates. After washing with soap and water by hand, they were cleaned 10 min each under sonication in water, ethanol, acetone, rotisol, ethanol and water. Finally, they were blow-dried with nitrogen. The glass chips were activated by treatment with oxygen plasma etching for 1 h at 380 W in a 200G Plasma System (TePla GmbH, Wettenberg, Germany) and afterwards directly transferred in a preactivated (10 min stirring) 1% APTES solution with 1 mM acetic acid for 10 min. A 5 min sonication in ultrapure water was the next step before the glass chips were again blow-dried with nitrogen and stored under an argon atmosphere or were used directly. On the respective APTES chips a droplet of 20 µl 10 × concentrated spherical 80 nm AuNP from BBI (British BioCell International, Cardiff, UK) were deposited and incubated for 1 h at room temperature. If coverslips were used, they were treated similar. The produced AuNP chips were then dipped two times in water for washing and carefully blow-dried with nitrogen. Stored in a closed container, the chips can be used from several month up to years.

### LSPR instrument

All microfluidic assays used a custom build LSPR instrument (figure S3) at IPHT-Leibniz. It consists of a halogen light source HL-2000-FHSA (Ocean Optics, USA), an optical fiber connected UV/VIS linear photodiode array spectrometer USB 2000+ (Ocean Optics, USA), a peristaltic pump (Ismatec Reglo-ICC, Cole-Parmer GmbH, Wertheim, Germany), a 2-way valve (Bio-Chem Fluidics Inc, Boonton, USA) for the waste and a custom designed 3D printed microfluidic chamber (figure S4) and sealing with two inputs and one output capillary at each channel side similar to an earlier published setup^28–30^. For some measurements single-use flow cells “Basic sensor platform II” (# 10001354, microfluidic chip shop GmbH, Jena, Germany) were used with adhesive tape gasket for Fl. 1005—rhombic chamber shape # 10001361. The in- and outputs were used in the same way as for the 3D printed chamber by blocking unused channels with a plug from the same company. For the pump and valve control, a custom-built Python program was used which also records the spectral information and calculates the centroid position of the LPSR peak in nm which is then visualized in a sensogram (plot of peak wavelength against time). The evaluation of the measured sensogram was also done via a Python script to extract mean values, standard deviations of mean values and wavelength shift (Δλ) values. Dried AuNP-Chips with or without SAM were attached to 3D printed microfluidic chamber with specific 3D printed sealing or glued in the commercial chamber. If the commercial chamber was used, it is mentioned in the method. All chips were cleaned before use (in the case of SAM before the SAM deposition) 3 min under ozone (UV ozone cleaner UVC-1014 Nano-BioAnalytics, Berlin, Germany). Before and after each measurement, the whole system was flushed with water for at least 15 min. All solutions used in the microfluidic system were filtered with a 0.22 µm Syringe filter (Carl Roth GmbH + Co. KG, Karlsruhe, Germany) and afterwards degassed for at least 30 min under vacuum (Air Admiral, Cole-Parmer GmbH, Wertheim, Germany) in a desiccator (Nalgen, Thermo Fisher Scientific, Waltham, USA). At the beginning of each measurement, a lamp spectrum without an AuNP-chip was recorded for background subtraction and noise reduction. The specific refractive indices of the used buffers were measured with a portable refractometer PAL-RI B331500 (ATAGO, Tokio, Japan) and entered into the software. Afterwards, a calibration script was used to calculate the bulk sensitivity of the chip by alternately flushing two different buffers with different refractive indices and recording the associated centroid wavelength. The bulk sensitivity (S_B_) is calculated according to Eq. 1:

1 $$ {\text{S}}_{{\text{B}}} = \Delta \lambda /\Delta {\text{n}}_{{\text{b}}} $$

where Δλ is the wavelength shift of the centroid position and Δn_b_ is the refractive index change for the specific buffer solutions. Three times the standard deviation of a mean over at least 50 s of buffer injection (using as reference or blank) was used as threshold value for the limit of detection (LOD) calculation.

### Layer by layer deposition

For a pure PAH/PSS assay without any CRP, both 1 mM PAH and 1 mM PSS (with respect to the monomer) were dissolved in 0.1 M NaCl, respectively. The solutions were pumped alternately over the AuNP-chip with a 100 s buffer (0.1 M NaCl) injection before and after each 150 s polyelectrolyte injection. The flowrates were set to 10 µl/min. The surface sensitivity (S_S_) is calculated according to Eq. 2:

2 $$ {\text{S}}_{{\text{S}}} = \Delta \lambda /\Delta {\text{n}}_{{\text{l}}} $$

where λ is the wavelength of the centroid positions (Δλ = λ_bilayer n_ − λ_bilayer n−1_) and n_l_ is the refractive index of the layer (Δn_l_ = n_bilayer_ − n_buffer_).

The decay length (l_d_) of the immobilized particles and the refractive index sensitivity (m) was calculated by plotting the recorded plasmon shifts (Δλ) against the layer thickness (d) and fitting the data with Eq. 3^31,32^. The layer thickness of a PAH/PSS bilayer in aqueous environment is ~ 4 nm^33^ and the refractive index of the bilayer is ~ 1.5 RIU except for the first two to three bilayer^34^. With respect to the refractive index of the 0.1 M NaCl buffer which was 1.3305 RIU, Δn = 0.1695 RIU.

Equation 3:

3 $$ \Delta \lambda = m*\Delta {\text{n}}_{{\text{l}}} *[1 - {\text{e}}^{ \wedge } ( - 2{\text{d}}/{\text{l}}_{{\text{d}}} )] $$

For the measurement of the CRP deposition on three or four PAH/PSS bilayers, coverslips and the commercial flow-chamber were used. Therefore, 2 mg/ml PAH, 2 mg/ml PSS and 31 µg/ml CRP each were dissolved in 0.5 M NaCl buffer. The PAH and PSS solutions were pumped alternately over the AuNP-chip with a buffer injection of 0.5 M NaCl before and after each polyelectrolyte injection up to 3 or 4 bilayers. Then the CRP was injected followed by another buffer step. The flowrates were set to 20 µl/min and all injection times were 150 s.

### CRP-assay design

For all the assay measurements, the flowrates were set to 10 µl/min except for capture and target solutions which were pumped with 5 µl/min. For a clear distinction between bulk signals and binding events, the same buffer was injected before and after all reagents. In general, NaAc-Buffer (pH 4; 4.5 or 5) was used for immobilization and 1 × PBS as running buffer (Supporting Table S1). While running the first step, the channel for the second step was already pre-flushed with 3 µl/min directly to waste as well as further steps analogously. This was ensured by injecting the solutions alternately from different sides of the chamber, and opening the corresponding outlet valves. Due to the pre-flow and alternating flow techniques, the selected solutions ran through the channel directly after switching to the corresponding step. The software records all the injection steps accurately. Due to a time resolution of the spectrometer in the second range (1.5–3 s per measuring point), kinetic measurements are also possible. After the regeneration, the chip could be used for further target injections or a negative control. All capture reagents and BSA were dissolved in immobilization buffer. Targets and negative controls were dissolved in running buffer.

### Chemical immobilization of α-hCRPc via EDC/NHS

Carboxyl-groups, which are required for EDC/NHS coupling, were realized on the AuNP-Chips with MUA as SAM. Therefore, the chips were incubated overnight in 1 mM MUA, 0.5 mM MUA with 5 mM MUD or 0.5 mM MUA with 5 mM 1-OT in ethanol to obtain different SAMs of MUA, MUA/MUD or MUA/1-OT on AuNP-Chips. MUA, MUD and 1-OT were purchased from Sigma (Sigma-Aldrich, Munich, Germany). Before using the chips, they were washed again shortly in ethanol and water. To identify the optimal pH for the immobilization buffer, 0.25 mg/ml α-hCRPc was dissolved in buffers with different pH and an immobilization pH scouting was carried out^35^. Therefore, the capture solutions pH 4, 4.5 and 5 were shortly pumped over the non-activated surface and electrostatically bound molecules were washed away with ethanolamine. The solution with the highest λ shift—pH 5—was chosen for the chemical immobilization. To obtain a covalent binding of the capture antibody to the AuNP-Chip, the carboxyl groups were activated by flushing 0.4 M EDC and 0.1 M NHS parallel with 5 µl/min from the same side in the chamber (finally 0.2 M EDC and 0.05 M NHS). After this activation, the capture solution was flushed over the surface for 400 s and the antibodies were bound randomly via their free amino groups (e.g., lysine). In the next step, the chip was flushed with ethanolamine for 500 s to wash away unspecific bound proteins and to react with the remaining activated carboxyl groups^36^. BSA blocking with 10 mg/ml in NaAc pH 4 for 300 s and a flow rate of 10 µl/min was still necessary.

### Thiol-streptavidin mediated immobilization of α-hCRPcb

Thiol modified streptavidin (SH-SA) was purchased from Protein Mods LLC (Waunakee, USA) and used as received without further purification or dilution. Blank AuNP-Chips can be easily functionalized with SH-SA by flushing a solution of 1 mg/ml over the surface for 200 s with 5 µl/min. In order to sufficiently block the sensor surface, various concentrations of BSA were tested in different buffers. Starting from 1 mg/ml over 10 mg/ml in PBS up to 1; 2 and 2.5 mg/ml in NaAc pH 4. Flow rates and injection times were also slightly varied between 5/10 µl/min and 200/250 s. After the blocking step a solution 0.25 mg/ml of biotinylated antibody α-hCRPcb was injected for 300 s with 5 µl/min. So, the chip was ready to use for hCRP detection without further blocking steps. For immobilization outside the chamber, one drop each of the SH-SA and the α-hCRPcb solutions were pipetted onto the AuNP chip, one after the other. Each of these were incubated for 1 h at 23 °C with 15–20% humidity, prior to washing 10 min with PBS (150 rpm horizontal shaking) and flushed with ultrapure water and dried in nitrogen stream (here called SH-SA outside).

## Results

### Signal and sensitivity dependency on layer thickness

**Figure 2:**
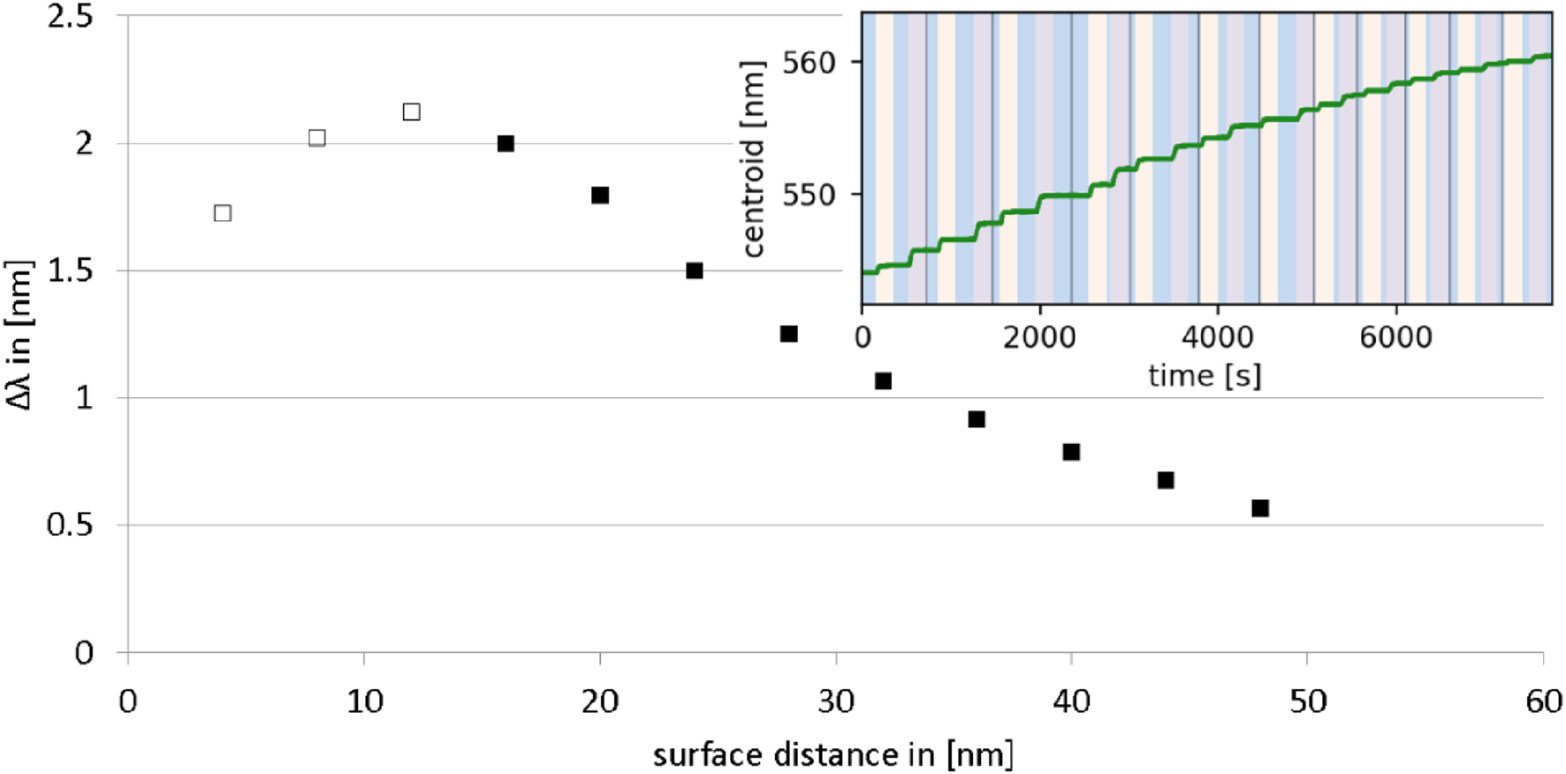
PAH/PSS bilayers realized by layer-by-layer (LbL) deposition. Plasmon shift (Δλ) in nm for each bilayer plotted against the number of PAH/PSS bilayer deposited on 80 nm spherical gold nano-particle sensors. The first two to three bilayers layers (unfilled squares) are known to be improperly formed^34^. Inset: Original sensogram plotting the LSPR response (peak wavelength) against time. Blue = 0.1 M NaCl, ivory = 1 mM PAH and purple = 1 mM PSS.

LSPR detection yielded the sensor response for attachment or binding events on the sensor surface. The well-established layer-by-layer deposition using PAH and PSS was utilized to demonstrate the sensing approach^34,37–39^: The deposition of each additional layer on a gold nanoparticle chip (80 nm spheres) in a microfluidic system resulted in a longer wavelength (red) shift of the resonance peak. This is visible as individual steps in the sensogram, which plots the LSPR response over time (Fig. 2 inset).

The first two to three bilayers were improperly formed due to a higher water content, resulting in lower refractive index and lower signal shifts than expected. After the 3rd bilayer, the refractive index of the bilayers was approximately 1.5^34^, the bilayer thickness in the wet state was approximately 4 nm^33^. The centroid of the LSPR-peak was measured over time and the shifts for each bilayer were plotted against the number of layers (Fig. 2). The evidence shows the signals for the bilayers decreased with increasing number of bilayers. This is due to a higher surface distance, where the field decay results in decreasing values. Also, the surface sensitivity was decreasing with increasing distance to the surface of the gold nanoparticles. With Eq. 3 the decay length (l_d_) of this AuNP-chip was calculated to be 79.5 nm with refractive index sensitivity (m) = 141.1 nm/RIU. The before measured bulk sensitivity (S_B_), calculated with Eq. 1 using the calibration, was with S_B_ = 115.0 nm/RIU, i.e. slightly lower. For 3 bilayers, the S_S_ was calculated to be 66.9 nm/RIU.

Although silver particles would provide better plasmonic properties, the higher chemical stability of gold results in a broader use of gold nanoparticles in LSPR sensing^40^. The spherical 80 nm gold nanoparticles therefore used in our investigations were developed over the years^28,41^ as a good compromise between larger (and therefore more sensitive, but less homogenous synthesis) and smaller (less sensitive but better reproducible regarding shape and size distribution) sized nanoparticles. Anisotropic shaped particles would provide higher sensitivity, but are more complex to synthesize and usually difficult to biofunctionalize, therefore they were not utilized in this study.

### CRP detection

#### Immobilization via thiolated streptavidin

It is well established that thiolated compounds form molecular layers on gold surfaces. One example are thiol-alkanes as well-studied model systems in the field of SAMs. On the other hand, biotin/(strept)avidin coupling is a powerful platform for nanoscale fabrication with many different applications in science, medicine, and nanotechnology. Combining these two well-established and straightforward attachment approaches, a scheme using thiolated streptavidin layers on the gold nanoparticle surfaces, and subsequent attachment of biotinylated antibodies on the SAMs, was utilized to prepare sensor substrates for CRP detection (Fig. 1 left).

**Figure 3:**
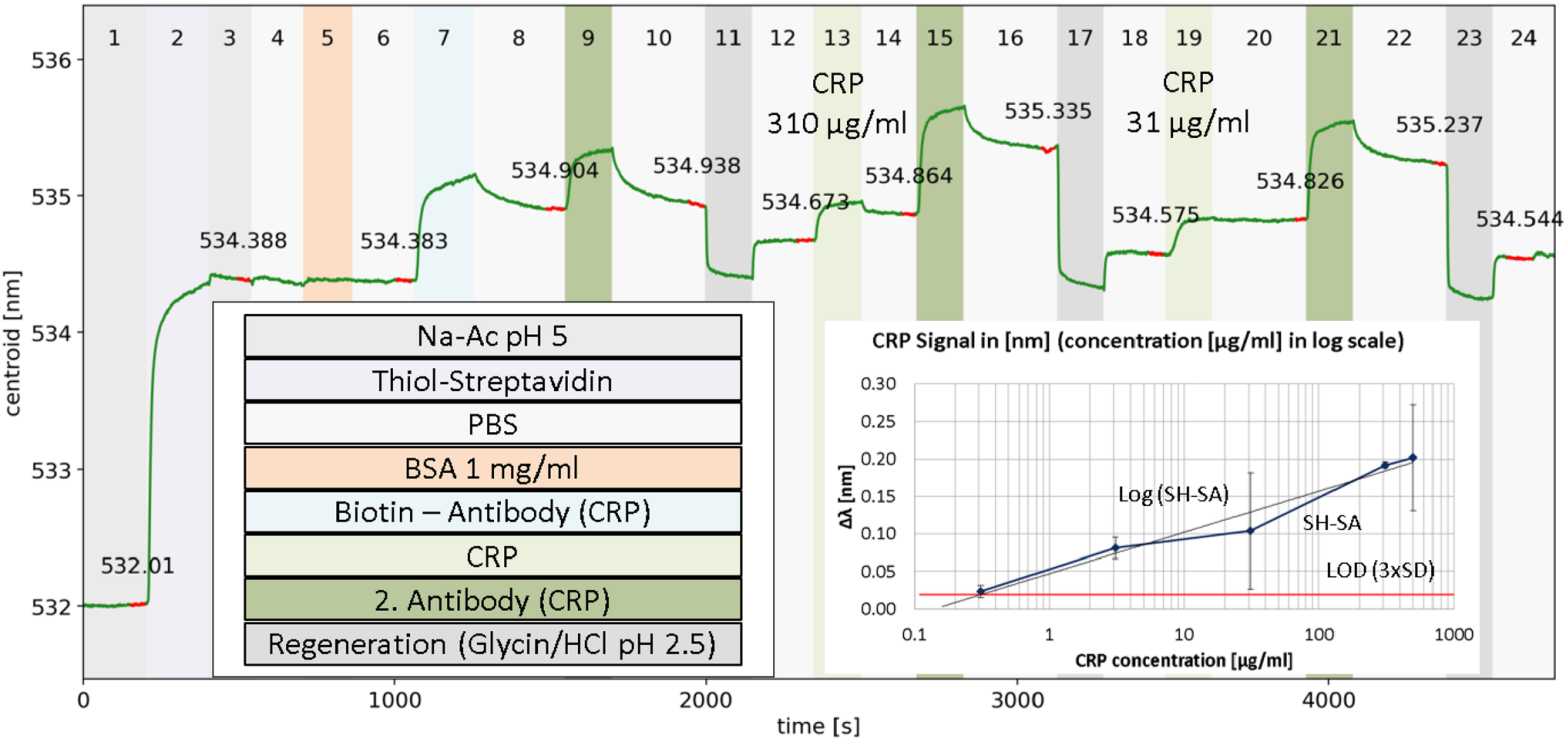
Example measurement for thiol-streptavidin mediated CRP binding assay. A substrate with gold nanoparticles modified by thiol streptavidin was utilized for attachment of CRP anti-bodies prior to binding of CRP. Inset right: Sensor response Δλ (mean values) in nm for 0.31; 3,1; 31, 310 and 500 µg/ml CRP. LOD is calculated with three times the standard deviation of the blank (PBS buffer).

The preparation and subsequent performance of these sensor substrates for CRP detection is documented in Fig. 3. In the beginning (1), immobilized gold nanoparticles in a 10 mM NaAc pH 5-filled fluid cell result in a localized surface plasmon resonance (centroid) of 532.01 nm. Then, the cell was flushed with thiol-streptavidin, the resulting significant increase in centroid wavelength (to 534.388 nm) indicates strong binding on the gold nanoparticle surface (2). After buffer washing steps (3,4) a 1 mg/ml BSA passivation (5) induced only a small signal shift of 0.033 nm, which pointed to a weak BSA absorption on the gold particles in PBS buffer. Now, after a wash step (6), the biotinylated antibody (α-hCRPcb) was binding (7) with a shift of 0.521 nm. Afterwards, again the buffer (8) was injected, and a negative control (9) was conducted by applying the secondary antibody (α-hCRPd), which should bind on CRP only in case of a successful capturing. Because no CRP was present at this moment, this secondary antibody had no specific binding partner. However, the LSPR signal increased, indicating the presence of the secondary antibody at the surface. However, the subsequent washing step (10) appeared to remove it completely, the signal decreased to the previous level (cf. levels at 8 and 10).

Next a regeneration step was introduced to check for—and remove—loosely bound molecules from the surface (11). The utilized solution seemed to have a lower refractive index as the standard buffer in 10, so the initial steep decrease in 11 can be attributed to this difference. However, afterwards the buffer from 10 was used again (12), allowed for a reproducible measurement: about 0.3 nm decrease between 10 and 12. Comparing 12 with 6 indicates that some of the attached biotinylated antibodies (7) were still on the substrate. After this preparation of the sensor surface, the actual CRP sensing step followed: 310 µg/ml CRP was flushed through the liquid cell leading to a measurable signal increase (13). This slightly decreased in a subsequent washing step (14), so that an overall CRP signal of about 0.2 nm resulted. Now, the already introduced secondary antibody was applied (15), testing on one hand the specificity of the CRP binding and, on the other hand, demonstrating possible signal amplification. There was a significant signal decrease of about 0.4 nm after a subsequent washing (16), which indicates a removal of some of the secondary antibodies. However, when CRP was flushed in again (19), this time one-tenth of the original concentration, the similar increase in signal was observed as before in step 13. This points to the fact that a saturation of the binding capacity was reached, even with the low concentration. A closer look at 13 and 19 reveals that the initial part of the curve was steeper in the case of higher concentration (13) compared to the lower one (19). When the secondary antibody was flushed in again (21), the same signal increase resulted as before in 15, indicating good reproducibility. This sensogram was selected to provide an example to highlight potential problems and challenges such as sensor regeneration, insufficient blocking or non-specific binding. Details will be discussed in the following paragraphs.

#### Limit of detection

Despite a significant lower response of SH-SA mediated CRP binding the measured limit of detection was 0.3 µg/ml (0.3 mg/L). With suitable regeneration solutions, the target molecules could be washed away, so that the sensor chip was available for another sample. Considering three times the standard deviation of a PBS buffer step (blank), the LOD was close to 0.3 µg/ml (Fig. 3 inset)^42^. In addition, the LOD could be improved by using signal amplification with secondary antibodies (sandwich immunoassay). Here, the secondary antibodies were used to evaluate the specificity of the assay. In an example measurement (Fig. 3), blocking with 1 mg/ml BSA in PBS was insufficient since unspecific binding of α-hCRPd in seen in step 9. But, as visible in step 10, the buffer was quickly washing away the “negative control” in the presence of CRP (steps 15/16 & 21/22). We assume that were close to, or above, the upper limit of quantification with the concentrations shown in Fig. 3 for CRP 1:10 (310 µg/ml). We support this claim with the observed shift for 310 µg/ml CRP being slightly lower than for 31 µg/ml CRP. The higher concentration still showed a higher shift during the injection (Step 13), but decreased relatively quickly in Step 14, which could indicate a saturation of the available binding sites. Another explanation for the decreased signal at higher concentrations could be the "hook effect", whereby the effectiveness of antibodies to form immune complexes is sometimes impaired when concentrations of an antibody or an antigen are very high. This can occur in sandwich immunoassays as well as in competitive format at high target concentrations and was also manifested by lower signal at higher concentrations^43^. However, such a high 31 µg/ml CRP signal as shown in Fig. 3 could not be confirmed with any other SH-SA assay. The other measurements were significantly lower, as shown in the comparison of the mean values in Fig. 5 and also in the LOD inset (Fig. 3). The regeneration with 10 mM glycine–HCl pH 2.5 also appeared to be too harsh, which was noticeable by the fact that the buffer steps showed slightly lower centroid signal after each regeneration (steps 11/12; 17/18 & 22/23). This problem could not be solved. Still after 5 or more regenerations the signal was stable, and the target shift was comparable.

#### Immobilization pH scout for EDC/NHS chemistry

The efficiency of a chemical coupling of a capture molecule to a SAM via EDC/NHS strongly depends on the electrostatic interaction between the molecule and the surface. The pH value of the used immobilization buffer should be preferably adjusted between the isoelectric point (pI) of the capture molecule and the pKa of the SAM^35^. In this way, an electrostatic concentration of the protein to be immobilized will take place on the SAM surface, ensuring optimal binding. It is known from the literature that the pKa of MUA SAMs on particles is significantly higher (pKa = 6.8 for particles of 5 nm diameter and pKa ≈ 10 for flat surfaces) than for MUA in solution (pKa = 4.8) and also increases with increasing particle size^44,45^. Even the concentration and the size of surrounding ions have an impact on the pKa of MUA on nanoparticles, as well as the presence of other SAM-forming molecules such as 1-OT or MUD^44^. The pI value of the α-hCRPc used was not known and therefore immobilization scouting was performed with different pH values (4, 4.5 or 5) in 10 mM NaAc buffer (Figure S1). 1 M ethanolamine hydrochloride pH 8.5 was used to remove electrostatically bound protein from the not yet activated MUA surface. The highest plasmon shift was visible at pH 5. This pH was therefore chosen for the final immobilization.

#### Immobilization via MUA and subsequent EDC/NHS chemistry

The results with the thiolated streptavidin-immobilization chemistry (reported in the previous section) yielded a stable and measurable signal for the studied relevant CRP concentrations. However, as the scheme in Fig. 1 shows, the resulting construct of streptavidin and anti-CRP antibody spans a significant distant away from the sensor surface. As demonstrated in the layer-by-layer adsorption experiments in Fig. 2, an increasing distance from the surface hampered the achieved signal. In order to address this shortcoming, another immobilization approach was considered, which would allow decreasing the distance of the binding target to the sensor surface. It is based on a MUA self-assembled monolayer, binding on one side via thiol to the gold, and providing the means for an EDC/NHS-attachment of the anti-CRP antibody on the other side (Fig. 1, right).

#### Blocking for EDC/NHS chemistry

**Figure 4:**
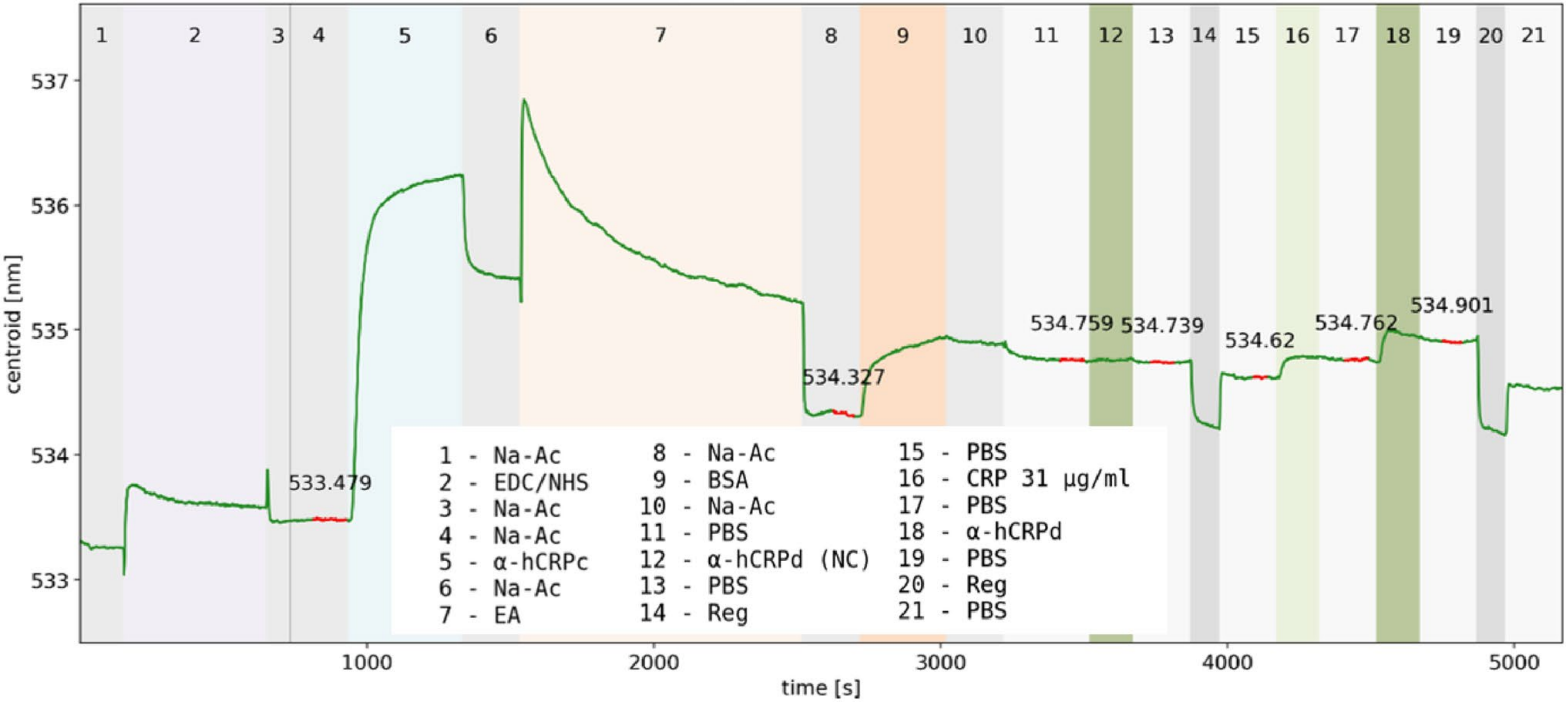
CRP assay on MUA/1-OT SAM with EDC/NHS chemistry. Step 9 is the 10 mg/ml BSA blocking and step 12 is a secondary anti CRP antibody with no CRP injection before as negative control. Reg = regeneration with 10 mM glycine–HCl pH 2.5.

A blocking step was essential to ensure that the "target" molecule under investigation was bound specifically to the immobilized captured molecule and not already adhered non-specifically to the sensor surface. In the initial SH-SA measurements with 1 mg/ml BSA in PBS as blocking reagent, nonspecific binding was clearly visible (Fig. 3). By increasing the BSA concentration to 10 mg/ml and changing the buffer to 10 mM NaAc pH 4, the blocking of the sensor surface was significantly improved. The step 9 in Fig. 4 showed a sufficient blocking with 10 mg/ml BSA, demonstrated by the secondary anti-human CRP antibody (α-hCRPd) as negative control in step 12, which showed no detectable unspecific binding. Only after CRP injection (step 16) was the α-hCRPd able to bind specifically, which resulted in a significant shift (step 18). On the other hand, the observed binding of α-hCRPd in the SH-SA assays (Fig. 3 step 9) could be due to a weak cross-reactivity to SH-SA. This would also explain why the sensogram course in Fig. 3 Steps 16 and 22 (dissociation of α-hCRPd after CRP injection) drops similarly as before CRP in Step 10. Such a dissociation course (obviously no 1:1 binding^46^) is not observed in the sensograms of the measurements without SH-SA (SAM method, Fig. 4 Steps 12/13 and 18/19). Since this effect was not investigated further, we chose the term ‘nonspecific’.

## Discussion

When considering a LSPR experiment for the detection of a target molecule one should consider the optimal immobilization method of the capture molecule. It depends on the (physico)chemical nature of the capture molecule (DNA, proteins, lipids and others) and the possible required modifications for its immobilization. Proteins, for example, are known to bind directly on bare gold surfaces. However, this can have an impact on their stability and functionality^30^ and can result in weakened target binding. Different immobilization methods have been developed in the past to address this challenge, as described above. Another important factor for an efficient target binding (and therefore signal strength) is the distance of the binding site from the surface. This parameter can be modulated by the choice of the immobilization chemistry, as highlighted in this study.

The PAH/PSS bilayer deposition experiments yielded an optimal range for the distance of the target molecules from the sensor's surface. For the AuNP chips used in this study, the first 24 nm from the surface would result in a significant shift (Δλ) of at least 1.5 nm for the binding of a well packed PAH/PSS bilayer with n_layer_ = 1.5 RIU. If the decay length (l_d_) would be higher, the signal shift for each bilayer would be smaller in comparison to shifts on particles with lower l_d_. On the other hand, the decrease of the signal shift for each following bilayer would also be smaller because the total sensing distance is higher, but the absolute maximum shift would be the same. For higher absolute maximum shift the increase of the refractive index sensitivity (m; m = S_B_ if d approaches to infinite^32^) would be necessary, which can be attained with bigger particles, other materials or particles with anisotropic shapes^32,47^. The calculated m and l_d_ with 141.1 nm/RIU and 79.5 nm for the utilized AuNP-chips differ slightly from the values expected by theory. This is reasonable because the refractive index of the layer and the layer thickness were taken from the literature. In the case of the CRP deposition, the measured plasmon shift was lower than 1.5 nm. This is confirmed in supporting Figure S2, 1.212 ± 0.032 nm/0.918 ± 0.012 nm on the 3rd/4th PAH/PSS bilayer corresponding to 12/16 nm away from the gold nanoparticle surface. Because of the water content the refractive index of a CRP layer is reported to be ≤ 1.45 RIU^48,49^ which is also reflected by a lower plasmon shift. It is observed that at least one bilayer more, approximately 4 nm, reduces the signal of the target significantly (by roughly 25%). After the 3rd PAH/PSS bilayer, the total centroid shift was 10.095 nm/10.607 nm, which indicates that the chips behave similarly having comparable surface and bulk sensitivities (S_S_ = 61.0 nm/RIU and 64.1 nm/RIU for three PAH/PSS bilayer; S_B_ = 97.87 nm/RIU and 115.09 nm/RIU).

The methods used for CRP detection in this work (SH-SA and SAM method) are based on covalent bonding to the gold surface. Both MUA (1-OT, MUD) and SH-SA have free thiol groups (-SH) that form a covalent Au–S bond at neutral to basic pH^50^. In the SH-SA method, antibody binding occurs via a biotin-streptavidin bond, which is one of the strongest non-covalent bonds in nature. In the SAM method, the antibodies are again covalently bound to the already anchored MUA via chemical immobilization. In this process, the free carboxyl groups first react with protonated EDC to form active O-acylisourea intermediates, which is then stabilized by NHS to form a succinimidyl ester^25^. Free amine side chains of the antibodies to be immobilized (e.g., lysine) can then react with the formed ester to create an amide bond with MUA^26^. This process is a zero-length crosslinking. Nevertheless, the orientation of the antibodies is most likely similarly random in both cases since the biotinylation of the antibodies also occurs via EDC/NHS in many cases. The binding of the antigen (CRP) then takes place via non-covalent bonds such as hydrogen bonding or hydrophobic interaction, which ensures the regenerability of the sensor^51^.

**Figure 5:**
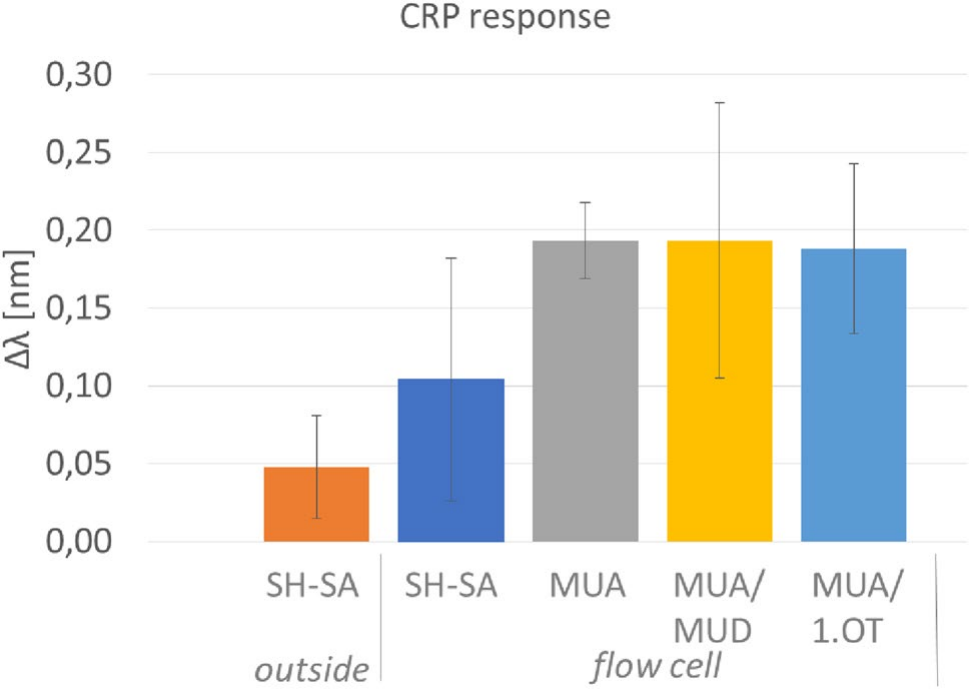
Sensor response Δλ in nm for 31 µg/ml CRP with differently immobilized capture antibody shown. Each value is a mean value of at least 3 independent measurements, error bars are standard deviations.

Using different immobilization methods for the capture antibodies, method-dependent differences are visible (Fig. 5). For the samples ‘SH-SA outside’, which was prepared outside of the fluid cell and air dried after antibody immobilization, a much smaller shift is observed, apparently air drying has a negative impact on the function of the detection antibody. However, for thiol-streptavidin (SH-SA) mediated immobilization, resulting in a greater surface distance, the plasmon shift for 31 µg/ml CRP was significantly lower than for the SAM method (independent samples T-test: p = 0.046; n ≥ 5 per group) which allows the target binding closer to the nanoparticle surface. On average, due to the thicker SH-SA layer, the target binding should be about 5 nm closer for the SAM method. On the other hand, it takes more time to produce the SAM chips (overnight), and also the EDC/NHS reaction is more time consuming during the assay. SH-SA immobilization and binding of the biotin capture antibody is straightforward and can be done completely in the microfluidic system. Blocking the surface against non-specific binding is necessary for both methods and could be sufficiently optimized for the assay. Regeneration of the sensor for antigen binding with 10 mM glycine HCl pH 2.5 was also successful. However, the LSPR signal after regeneration was always somewhat lower than before antigen binding, pointing to partial removal and/or inactivation of capture molecules, which indicates a need for optimization. The scatter of the CRP response was quite large. This could be due to less homogenous distribution of the AuNP including aggregations.

This is also reflected in the bulk sensitivities (S_B_) observed during calibration, as they showed a large variance (70.5–225.3 nm/RIU). However, as can be seen from the LbL data, this value cannot be equated with the calculated S_B_. Also, the measured surface sensitivities (S_S_) of the AuNP chips show a significantly lower variance (61.0–66.9 nm/RIU after 3 bilayer of PAH/PSS). With the detection limit of 0.3 mg/L CRP for SH-SA mediated immobilization, this immobilization method is fully sufficient for clinical applications and allows the measurement of several samples in one assay. When further improvement in sensitivity is required, the short-thiol mediated approach could increase the detection limit, but requires a more cumbersome preparation.

## Conclusions

The effect of the thickness of the capture probe attachment layer on the LSPR signal has been characterized. Using plain gold nanoparticle chips the straightforward SH-SA method is sufficient to determine CRP in clinically relevant concentrations. The immobilization layer is significantly thicker than with short thiols and thus also provides lower target signals. Using LbL technology, distance layers comparable to both studied immobilization chemistries could be prepared and compared regarding their LSPR signal shift upon binding of detection antibodies. The regenerability of the used sensors and the high time resolution of the utilized setup enables versatile applications such as diverse binding kinetic studies. The studied detection method is well suited for the discernment of protein biomarkers such as CRP in clinical applications allowing the measurement of several samples in one assay.

## Supplementary Information

Supplementary Information.

## Data Availability

The datasets generated during and/or analyzed during the current study are available from the corresponding author on reasonable request.
