# Supplementary material for: The effect of layer thickness and immobilization chemistry on the detection of CRP in LSPR assays
Source: Sci Rep. 2022 Jan 17;12:836. doi: 10.1038/s41598-022-04824-9 (PMC8763948; doi:10.1038/s41598-022-04824-9)
Supplement: Supplementary file 1 — Supplementary Information. [file 41598_2022_4824_MOESM1_ESM.docx]

Supporting Information

The effect of layer thickness and immobilization chemistry on the detection of CRP in LSPR Assays

Stephan Kastner, Pia Pritzke, Andrea Csáki and Wolfgang Fritzsche*

Department Nanobiophotonics, Leibniz Institute of Photonic Technology (IPHT), Jena, Germany

* wolfgang.fritzsche@leibniz-ipht.de

**Table S1:** Example CRP-Assay pump plan

| Step number | Comment | Reagent | Flowrate [µl/min] | time [s] |
| --- | --- | --- | --- | --- |
| 1 | immobilization buffer | 10 mM NaAc pH 5 | 10 | 200 |
| 2 | capture solution | 0.25 mg/ml α-hCRPc | 5 | 400 |
| 3 | immobilization buffer | 10 mM NaAc pH 5 | 10 | 200 |
| 4 | blocking solution | 10 mg/ml BSA | 10 | 300 |
| 5 | immobilization buffer | 10 mM NaAc pH 5 | 10 | 200 |
| 6 | running buffer | PBS | 10 | 300 |
| 7 | target solution | 31.2 µg/ml hCRP | 5 | 150 |
| 8 | running buffer | PBS | 10 | 300 |
| 9 | secondary antibody | 0.25 mg/ml α-hCRPd | 5 | 150 |
| 10 | running buffer | PBS | 10 | 300 |
| 11 | regeneration solution | 10 mM glycine-HCl pH 2.5 | 10 | 150 |
| 12 | running buffer | PBS | 10 | 300 |


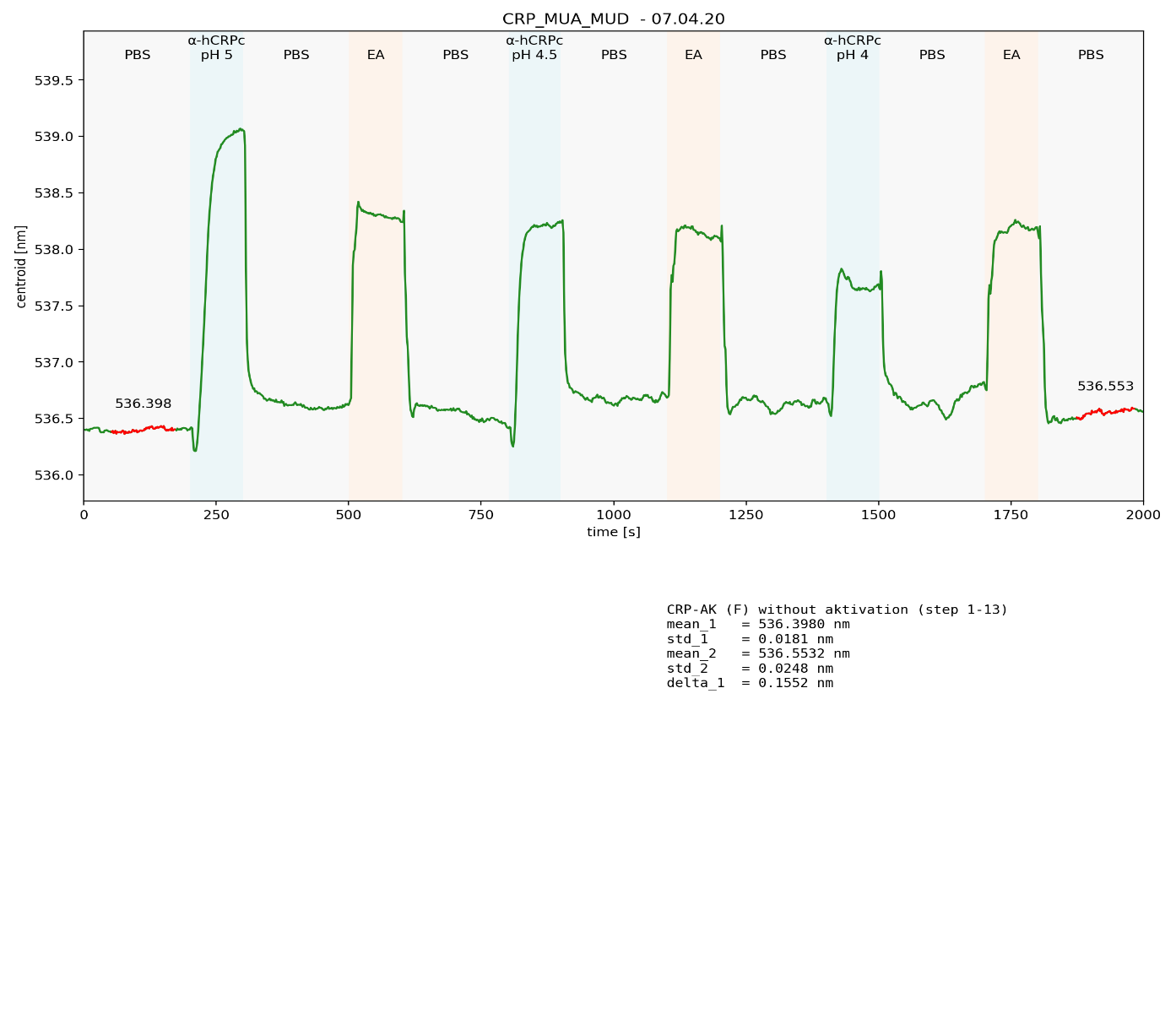


Figure S1

Immobilization scouting (testing various conditions for optimal binding) for EDC/NHS based anti-CRP antibody immobilization on MUA/MUD mixed SAM. Anti-human CRP antibody (α-hCRPc) in 10 mM NaAc buffer with varying pH (pH 4; 4.5 & 5) were compared to find best conditions. PBS = phosphate buffered saline; EA = 1 M ethanolamine hydrochloride pH 8.


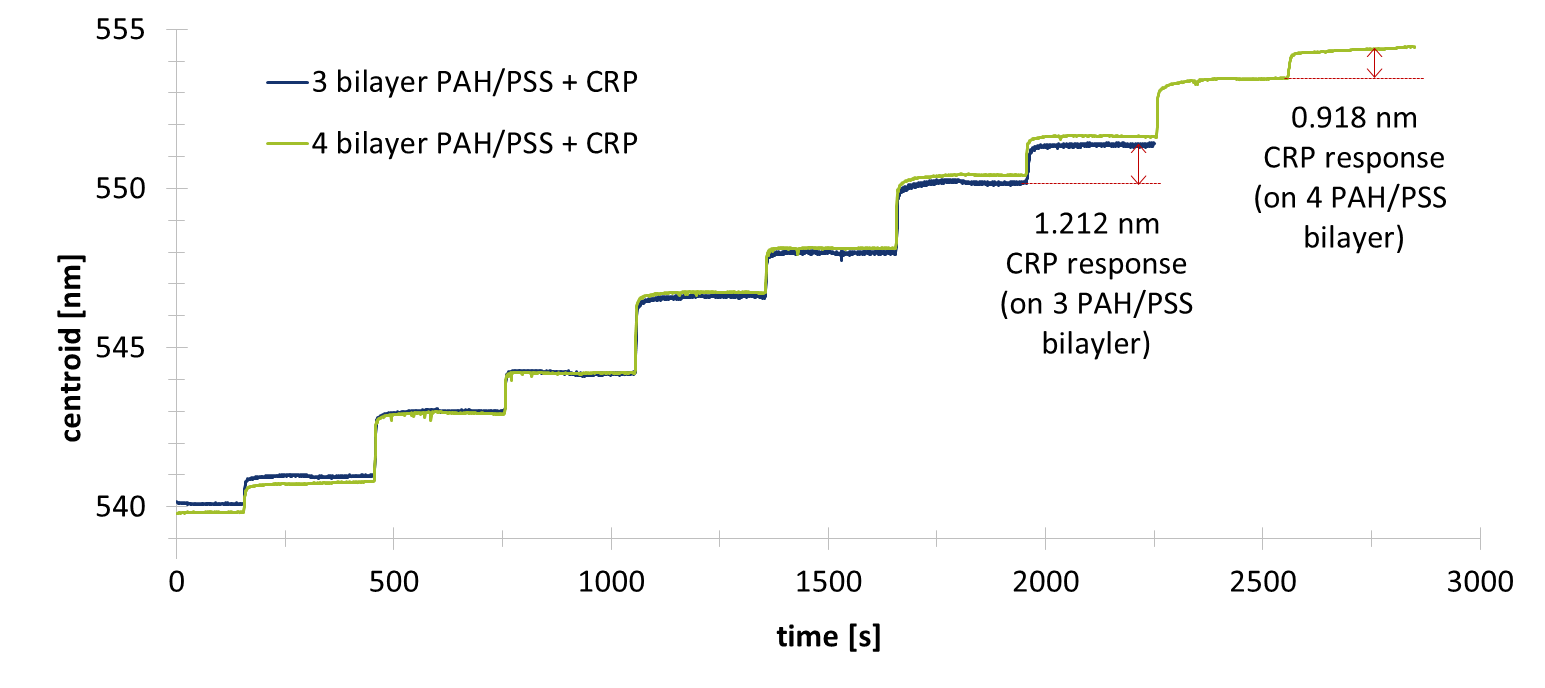


Figure S2

Sensogram of the deposition of 3/4 bilayers PAH/PSS prior to 31 µg/ml CRP. NaCl = 0.5 M sodium chloride; PAH = 2 mg/ml polyallylamine hydrochloride; PSS = 2 mg/ml polystyrene sulfonate; CRP = 31 µg/ml C-reactive protein; all in 0.5 M NaCl.


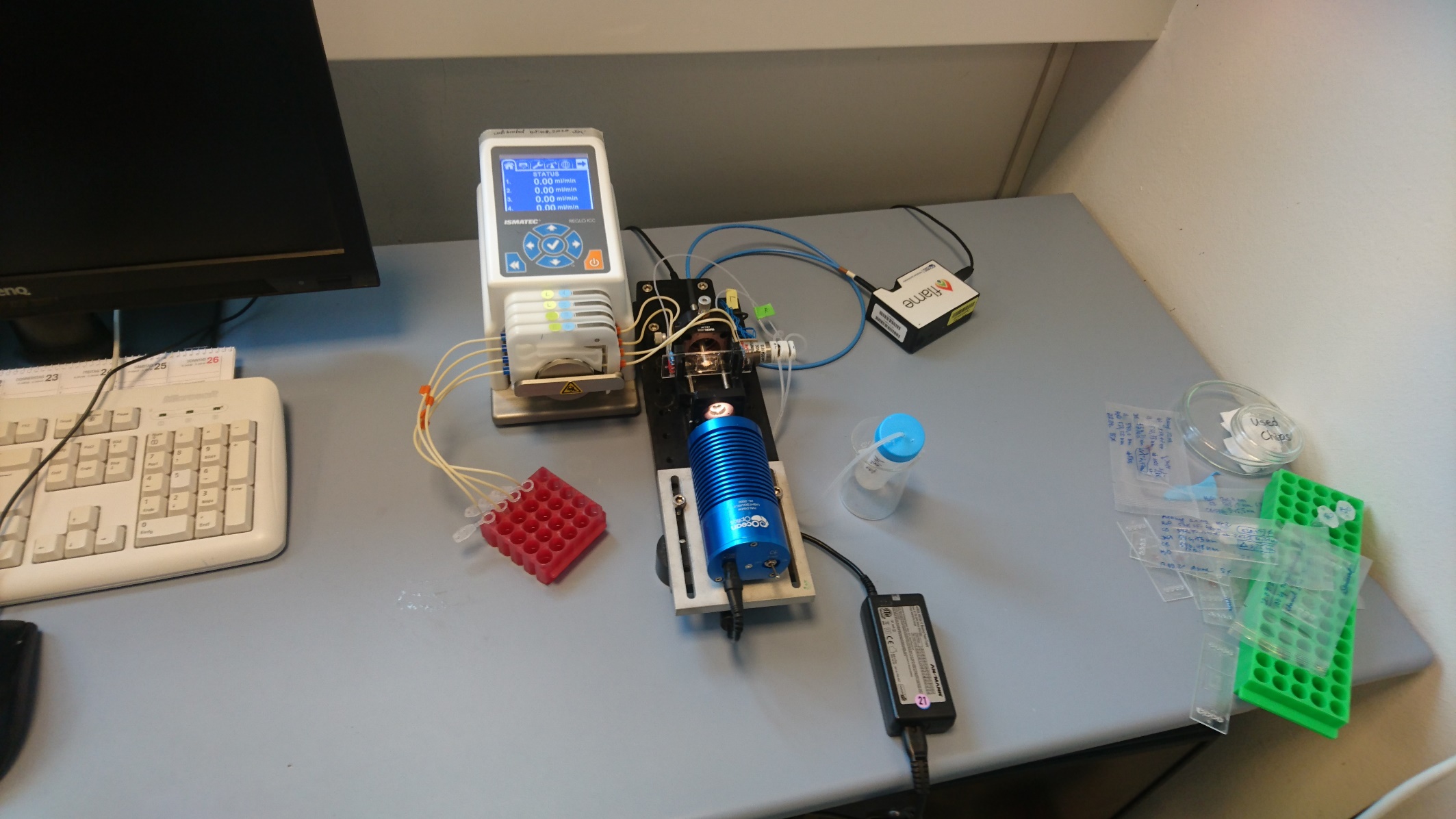


Samples

AuNP sensor

in flow cell

Halogen

light source

Fiber connected

spectrometer

Waste

Peristaltic pump

(4 individual

channels)

Figure S3

Picture of the instrumental setup with commercial chip chamber.

Figure S4

Picture of 3D printed chip chamber (right capillaries are not connected to the flow cell and were not used, only left capillaries were used). For each side of the flow cell there were three capillaries, two used as inlets connected to the pump and one as outlet to the waste.
